# Yes-associated protein reacts differently in vascular smooth muscle cells under different intensities of mechanical stretch

**DOI:** 10.18632/aging.203768

**Published:** 2022-01-04

**Authors:** Xinhao Wang, Xiangdang Liang, Lujun Zhou, Shen Liu, Zhuoqun Fang, Chuanzhong Hu, Yigong Hou, Zhanshe Guo

**Affiliations:** 1 Department of Vascular and Endovascular Surgery, Chinese PLA General Hospital, Beijing, China; 2 Department of Orthopedics, Chinese PLA General Hospital, Beijing, China; 3 State Key Laboratory of Brain and Cognitive Sciences, Institute of Biophysics, Chinese Academy of Sciences, Beijing, China; 4 School of Instrument Science and Optoelectronic Engineering, Beihang University, Beijing, China

**Keywords:** Hippo pathway, YAP, mechanical stretch, proliferation, apoptosis

## Abstract

Vascular smooth muscle cells (VSMCs) are stromal cells of the vascular wall and are continually exposed to mechanical signals. The loss of VSMCs is closely related to the occurrence of many vascular diseases, such as aortic aneurysms and aortic dissection. The proliferation and apoptosis of VSMCs are mechanically stimulated. Yes-associated protein (YAP), one of the core components of the Hippo pathway, plays a key role in the response of VSMCs to mechanical signals. In this study, we tested the impact of different intensities of mechanical stretch on the proliferation and apoptosis of VSMCs, as well as YAP. We tested VSMCs’ proliferation and apoptosis and YAP reaction via immunocytochemistry, western blotting, CCK-8 and flow cytometric analysis. We found that 10% elongation could increase the phosphorylation of YAP and prevent it from entering the nucleus, as well as inhibit cell proliferation and promote apoptosis. However, 15% elongation reduced YAP phosphorylation and promoted its nuclear entry, thereby promoting cell proliferation and inhibiting apoptosis. Accordingly, YAP knockdown suppressed the phenotype of VMSCs induced by 15% elongation. Taken together, YAP regulates proliferation and apoptosis of VSMCs differently under different intensity of mechanical stretch. Mechanical stretch with appropriate intensity can promote the proliferation and inhibit apoptosis of VSMCs by activating YAP.

## INTRODUCTION

Aortic aneurysms are associated with aortic dissection and rupture. There is currently no effective treatment to prevent or cure aortic aneurysms. At present, both the pathogenesis and pathophysiology of ascending aortic aneurysms are not entirely clear. Vascular smooth muscle cells (VSMCs) have been recognized as the most important factor in the development of ascending aortic aneurysms. Aortic aneurysms can occur due to loss of VSMCs in the media layer of the aortic wall, leading to progressive aortic dilation [1–3]. We observed an interesting phenomenon in clinical work where the aneurysm or dissection remodeling varies from site to site, which may be due to differences in the mechanical stimuli to which different sites are exposed.

Aortic walls are subjected to various mechanical stimuli from the bloodstream, such as shear and mechanical stretch. Stretch sensing is generally known as an integrin-mediated pathway, which is coupled to cell contractile activity, and thus shares many mechano-transduction pathways with the rigidity sensing process in translating mechanical stimuli into intracellular biochemical signals [4–6]. The relationship between mechanical stretch and cell proliferation/apoptosis has been extensively studied [7–11]. VMSCs experience mechanical stimuli during growth and differentiation and transduce these stimuli into biochemical signals that in turn regulate cell responses to the imposed forces. The effect of cyclic stretch is recognized as an important regulator of the development and pathological abnormalities of aortic walls. Under physiological conditions, the aorta undergoes approximately 10% circumferential stretch during systole. This number increases to approximately 20% under conditions of hypertension. The rate of apoptosis of VSMCs under 20% circumferential stretch is higher than that under 10% stimulation; how VSMCs change under 10–20% stretch remains uncertain [12].

The Hippo pathway plays an important role in the cell’s reaction to mechanical stretch [13–17]. It was first defined in *Drosophila* by genetic mosaic screening following identification of a loss-of-function mutation of Hippo that led to a strong overgrowth phenotype [18]. As the major downstream effector of the Hippo pathway, YAP/TAZ mediates major physiological functions therein. MST1/2, Sav1, LATS1/2, and Mob1 constitute a kinase cascade that eventually phosphorylates YAP/TAZ and promotes its binding with 14-3-3 and cytoplasmic retention [19, 20]. YAP/TAZ has been identified as the sensor and mediator of mechanical cues arising according to the rigidity of the extracellular matrix, cell geometry, cell density, and the status of the actin cytoskeleton [14, 15, 21, 22]. Among them, YAP, as an important factor in mechanical signal transduction, controls cell survival and proliferation by combining with DNA-binding transcription factors to induce gene expression (Hippo pathway in organ size control, tissue homeostasis, and cancer). YAP inhibits the expression of smooth muscle differentiation genes, and at the same time promotes smooth muscle proliferation and migration *in vitro* and *in vivo*, and plays a novel comprehensive role in smooth muscle phenotype regulation (The induction of yes-associated protein expression after arterial injury is crucial for smooth muscle phenotypic modulation and neointima formation). Therefore, these evidences indicate that YAP is a key molecule in the regulation of VSMC phenotype. Rho-ROCK is the signaling pathway upstream of Hippo. The Rho-ROCK signaling pathway inhibits Hippo pathway activity [23, 24]. Rho-ROCK is affected by mechanical stress and regulates the proliferation of VSMCs. However, the influence of different intensities of mechanical stretch on the Hippo pathway, and the role of Rho-ROCK in this mechanism, remain unclear.

In our study, we placed VSMCs under different intensities of mechanical stretch *in vitro*. We aim to find out that how the Hippo pathway and cell proliferation of VSMCs changes under mechanical stretch from 0% to 15% elongation. Based on our study, we wish to achieve a better understanding of the relationship between different intensities of mechanical stretch and cell proliferation of VSMCs.

## MATERIALS AND METHODS

### VSMCs isolation and culture

Sprague-Dawley rats (male, 200-250 g) were purchased from the animal experimental center of Academy of Military Medical Sciences of PLA (Beijing, China) and housed under the specific pathogen-free (SPF) conditions (temperature, 23 ± 2° C; relative humidity, 65% ± 5%; 12h / 12h light / dark cycle, 07:00-19:00) with free access to food and water for 3 days. SD rats were anesthetized with isoflurane, and then VSMCs were isolated from thoracic aorta using the explanting technique [25]. and cultured in Dulbecco’s modified Eagle’s medium (DMEM; Gibco, Grand Island, NY, USA) containing 10% fetal bovine serum (FBS; Gibco, Grand Island, NY, USA), 100 U/mL penicillin, and 100 μg/mL streptomycin at 37° C in a humidified atmosphere of 5% CO_2_. The medium was changed every 2 d, and cells were passaged by treatment with a 0.05% trypsin-EDTA solution. The cells were used between passages 3 to 8. All animal experimental procedures were in accordance with the National Institutes of Health's Guide for the Care and Use of Laboratory Animals and were approved by the Animal Care and Use Committee of the Military Medical Sciences of PLA, as well as the Animal Laboratory Administration Center and Ethics Committee of the Military Medical Sciences of PLA.

### Cyclic stretch stress on VSMCs

VSMCs were plated in 6-well plates (Flexcell International Corp., Hillsborough, NC, USA) coated by type I collagen (Solarbio, Beijing, China) at a concentration of 3 × 10^5^ cells/mL. After 24 h attachment, the cells were synchronized by DMEM with 10% FBS for another 24 h and then applied to cyclic stretch produced by FX-5000T Tension System (Flexcell International Corp., Hillsborough, NC, USA) with 10, 15, and 20% elongation at a frequency of 1 Hz (60 cycles/min), and the duration of cyclic stretching forces for 24 h.

### YAP siRNA

Experimental 1 consisted of the following groups: VSMCs, VSMCs+YAP siRNA NC (TTCTCCGAACGTGTCACGT), VSMCs+YAP siRNA 1 (ACAGCAGGAGTTATTTCGG), VSMCs+YAP siRNA 2 (GACCTCTTCTGGTCAGAGA), and VSMCs+YAP siRNA 3 (ATCACAATGATCAGACAAC). Cells were inoculated in 6-well plates at 2 × 10 ^6^ cells/well and cultured overnight. Each well was diluted to 250 μL of Opti-MEM I Reduced Serum Medium by adding 100 pmol of siRNA. Lipofectamine 2000 (5 μL; Life Technologies, Carlsbad, CA, USA) was added to 250 μL of Opti-MEM I Reduced Serum Medium and incubated for 5 min. Then, the siRNA solution was added to the Lipofectamine 2000 solution and incubated for 20 min. The culture medium in the cell culture plate was aspirated and 1.5 mL fresh medium was added. The siRNA solution was added to the samples and cultured in a 5% CO_2_ incubator at 37° C for 48 h. Western blotting was used to assess the results and VSMCs were reclassified according to the obtained results.

Experimental 2 consisted of the following groups: VSMCs control+0% elongation, VSMCs YAP shRNA+0% elongation, VSMCs control+10% elongation, VSMCs YAP shRNA+10% elongation, VSMCs control+15% elongation, and VSMCs YAP shRNA+15% elongation. shRNA using the best one of the previous experiment which is siYAP1. VSMCs were cultured as described above. Cells were infected with the viruses at a multiplicity of infection of 50, and control groups were transfected with lentiviruses containing control sequences.

### Inhibition of the Rho/ROCK pathway

Experimental 3 consisted of the following groups: VSMCs+0% elongation, VSMCs+0% elongation+Y27632, VSMCs+10% elongation, VSMCs+10% elongation+Y27632, VSMCs+15% elongation, and VSMCs+15% elongation+Y27632. VSMCs were cultured as described above. The stretching frequency was 1 Hz and the stretching times were set to 1 h and 6 h. The final concentration of Y27632 (3μmol/L; T1870; TargetMol, Boston, MA, USA) treatment was 50 μM (0.5% dimethyl sulfoxide concentration).

### Flow cytometric analysis and CCK-8 assay

VSMCs from each group were stained with annexin V-fluorescein isothiocyanate to determine the number of apoptotic cells. And another samples were fixed with 75% ethanol and treated with RNase to analyze the cell cycle. Then, cell nuclei were stained with propidium iodide (Molecular Probes, Eugene, OR, USA), and VSMCs were analyzed using a FACSCalibur flow cytometer and Cell Quest software (Becton Dickinson, Franklin Lakes, NJ, USA).

VSMCs were inoculated in 6-well culture plate. Cell counting kit-8 (CCK-8; DOJINDO, Kumamoto, Japan) solution was mixed with serum-free medium in a 1:10 ratio (v/v). The mixture was added at 100 μL per well and incubated at 37° C and 5% CO_2_ for 1 h. Absorbance at 450 nm was measured using an enzyme marker.

### Immunocytochemistry analysis

VSMCs from each group were fixed in 4% paraformaldehyde for 20 min, and permeabilized with 0.2% Triton X-100 for 10 min at room temperature. Each sample was dripped with 3% BSA blocking solution and sealed at room temperature for 30 min. Then, samples were incubated with primary antibody to YAP (bs-3605R; Bioss, Los Angeles, CA, USA) and fluorescent CY3 goat anti-rabbit IgG secondary antibody (rhodamine-labeled, BA1036; Boster Biological Technology, Pleasanton, CA, USA). The nuclei were counterstained with 4’, 6-diamidino-2-phenylindole (DAPI; 1:500). The samples were observed under the confocal laser scanning microscopy.

### Western blotting

Treated VSMCs were harvested in lysis buffer using protease inhibitors, and total protein was extracted and quantified using a BCA protein concentration kit according to the manufacturer’s instructions. Proteins were separated by sodium dodecyl sulfate polyacrylamide gel electrophoresis (SDS-PAGE) and transferred to polyvinylidene fluoride (PVDF) membranes. The membranes were blocked with 5% BSA in Tris-buffered saline with Tween-20 (TBST) for 2 h at room temperature, and incubated overnight at 4° C with primary antibody (anti-YAP [bs-3605R; Bioss], anti-p-YAP [ab76252; Abcam, Cambridge, UK], anti-cyclin D1 (ccnd1) [ab134175; Abcam], anti-Lats [ab243656; Abcam, Cambridge, UK], anti-p-Lats [bs-4082R; Bioss], anti-Rho [ab32046; Abcam, Cambridge, UK], anti-ROCK [ab45171; Abcam, Cambridge, UK], and anti-β-actin [bs-0061R; Bioss]). The membranes were incubated with IRDye 800CW goat anti-rabbit IgG (H+L) (926-32211; Licor, Lincoln, NB, USA) at room temperature for 1 h. Bands were detected using the chemiluminescent imaging system.

### Statistical analysis

One-way analysis of variance was performed in SPSS software (ver. 19.0; SPSS Inc., Chicago, IL, USA) to evaluate group differences. Data are expressed as means ± standard deviation, and *P* values < 0.05 were considered to indicate statistical significance.

### Availability of data and materials

All data generated or analyzed during this study are included in this published article.

## RESULTS

### Mechanical stretch intensity influences the proliferation and apoptosis of VSMCs

**Figure 1:**
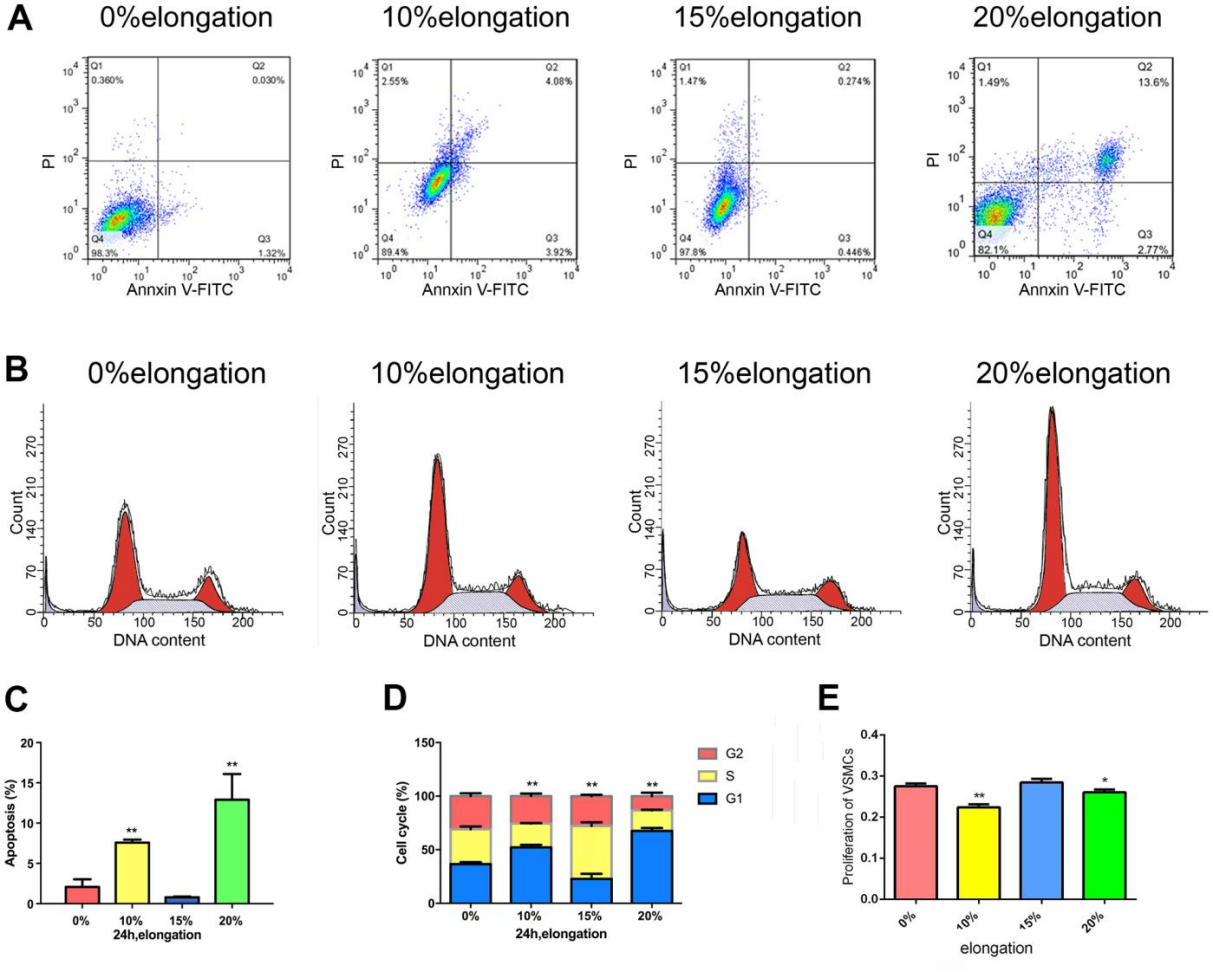
**Mechanical stretch intensity influences the proliferation and apoptosis of VSMCs.** (**A**) Apoptosis of VSMCs was detected by flow cytometry. (**B**) The cell cycle of VSMCs was detected by flow cytometry. (**C**) Quantitative analysis of apoptosis in VSMCs. (**D**) Quantitative analysis of the cell cycle in VSMCs. (**E**) The proliferation of VSMCs was detected by Cell Counting Kit 8 (CCK-8). Values are expressed as means±SD. ^*^P < 0.05, ^**^P < 0.01, compared with 0% elongation group.

To determine the changes in proliferation and apoptosis of VSMCs under different intensities of mechanical stretch, we applied 0, 10, 15, and 20% elongation forces to VSMCs (Figure 1). We used the CCK-8 assay to assess cell proliferation and flow cytometric analysis to detect apoptosis and analyze the cell cycle. The 10% and 20% elongation forces increased the rate of apoptosis and downregulated VSMCs proliferation (Figure 1). Meanwhile, 15% elongation produced the opposite results, i.e., decreased the rate of apoptosis and upregulated VSMCs proliferation (Figure 1). 10% and 20% of the stretch-induced S phase cells reduce might inhibit cell proliferation, while 15% stress-induced S phase cells increase might promote cell proliferation (Figure 1).

### Effects of different intensities of mechanical stress on YAP

**Figure 2:**
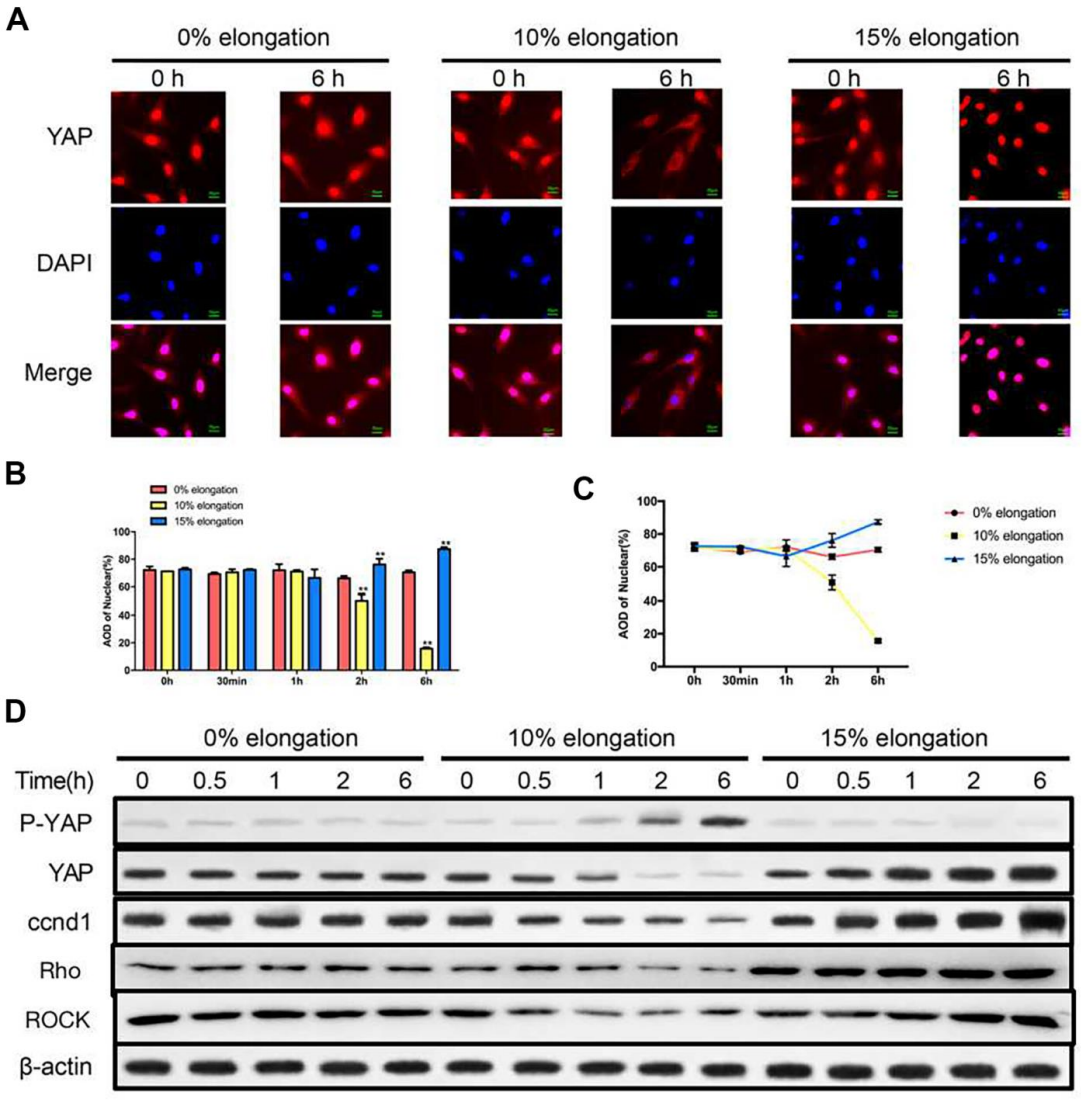
**Effects of different intensities of mechanical stress on YAP.** (**A**) The YAP localization of VSMCs was detected by immunocytochemistry. (**B**, **C**) Quantification of nuclear YAP amount in VSMCs. (**D**) The levels of phosphorylated YAP and cyclin D1 (ccnd1) in VSMCs were detected by western blotting. Values are expressed as means±SD. ^**^P < 0.01, compared with 0% elongation group.

To test whether YAP was involved in the effects of mechanical stress on VSMCs, we first performed immunofluorescence staining to determine the intracellular localization of YAP. YAP was both in the nucleus and the cytoplasm under 0% elongation (Figure 2A–2C). YAP was primarily cytoplasmic under 10% elongation conditions, whereas YAP was primarily nuclear with 15% elongation (Figure 2A–2C). Intracellular localization of YAP was linked to YAP phosphorylation, so we determined the level of YAP phosphorylation in each group. YAP phosphorylation was upregulated under 10% elongation, while phosphorylation of YAP remained low under both 0% and 15% elongation and being the lowest under 15% elongation (Figure 2D). Because nuclear localization of YAP was linked to the expression of ccnd1, we also assessed the protein levels of ccnd1 in each group at the same time. The protein levels of ccnd1 varied according to the phosphorylation level of YAP. Compared with 0% elongation, expression of ccnd1 decreased with 10%, and increased with 15% elongation (Figure 2D). Next, we tested the expression of key protein molecules in the Rho-ROCK signaling pathway. Similarly, compared with 0% elongation, expression of Rho and ROCK decreased with 10%, and increased with 15% elongation (Figure 2D).

### Knockdown of YAP impairs proliferation and apoptosis of VSMCs under different intensities of mechanical stretch

**Figure 3:**
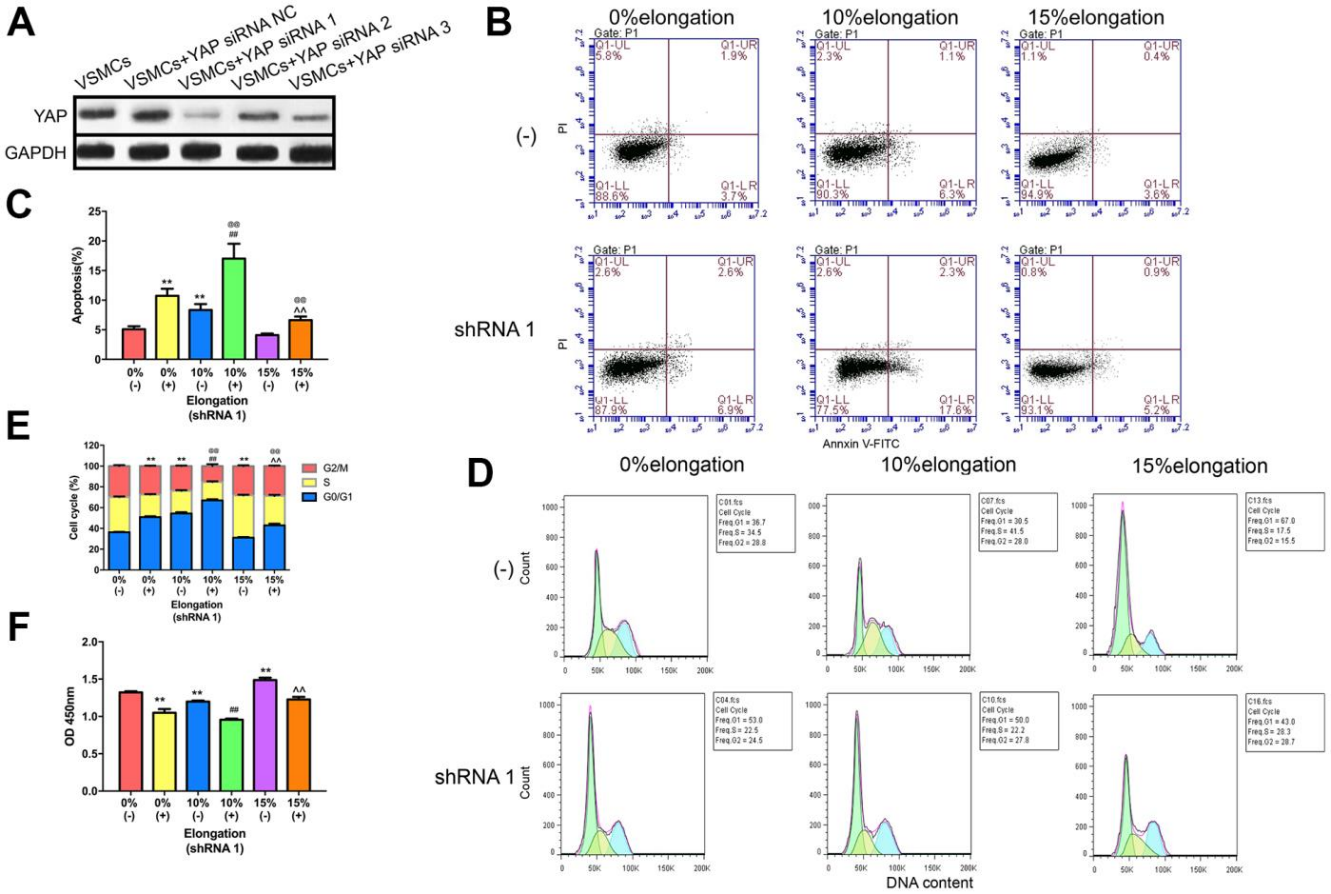
**Knockdown of YAP impairs proliferation and apoptosis of VSMCs under different intensities of mechanical stretch.** (**A**) The level of YAP expression in VSMCs transfected with YAP small interfering RNA (siRNA). (**B**) Apoptosis of VSMCs was detected by flow cytometry. (**C**) Quantitative analysis of apoptosis in VSMCs. (**D**) The cell cycle of VSMCs was detected by flow cytometry. (**E**) Quantitative analysis of the cell cycle in VSMCs. (**F**) The proliferation of VSMCs was detected by CCK-8. Values are expressed as means±SD. ^**^P < 0.01, compared with 0% elongation+siRNA(-) group; ^##^*P* < 0.01, compared with 10% elongation+siRNA(-) group; ^^^^*P* < 0.01, compared with 20% elongation+siRNA(-) group; ^@@^*P* < 0.01, compared with 0% elongation+siRNA(+) group.

To determine the effects of the Hippo pathway in VSMCs, we knocked down YAP by siRNA. We tested the efficiency of different YAP-targeting siRNAs using western blotting to generate optimal knockdown of YAP. Our results showed that YAP siRNA1 (ACAGCAGGAGTTATTTCGG) resulted in the greatest knockdown of YAP (Figure 3A). To test the effects of YAP knockdown on VSMCs proliferation, apoptosis, and the cell cycle under different intensities of mechanical stimulation, we performed CCK-8 assays and flow cytometry. The CCK-8 assays indicated that cell proliferation was inhibited following YAP knockdown in each group (Figure 3F). Compared with 0% elongation, VSMCs proliferation was decreased in the absence of YAP with 10% elongation, but increased with 15% elongation (Figure 3F). Following YAP knockdown, VSMCs showed a similar rate of proliferation under the different mechanical stimulations, but with smaller amplitudes (Figure 3F). Flow cytometry analysis demonstrated that apoptosis of VSMCs was increased following YAP knockdown under all stretch intensities (Figure 3B, 3C). The rate of apoptosis of VSMCs was significantly increased following YAP knockdown under 10% elongation (Figure 3B, 3C). Moreover, flow cytometry revealed that YAP knockdown kept more VSMCs in the G0/G1 phase under the same intensity of stretch, indicating that more VSMCs were in a state of dormancy (Figure 3D, 3E). Compared with the 0% control group, G0/G1 phase cells in the 10% and 15% control group were significantly increased and decreased, respectively (Figure 3D, 3E). A similar trend was apparent in the YAP shRNA groups (Figure 3D, 3E).

### Inhibition of the Rho-ROCK pathway affects the Hippo pathway under different intensities of stretch

**Figure 4:**
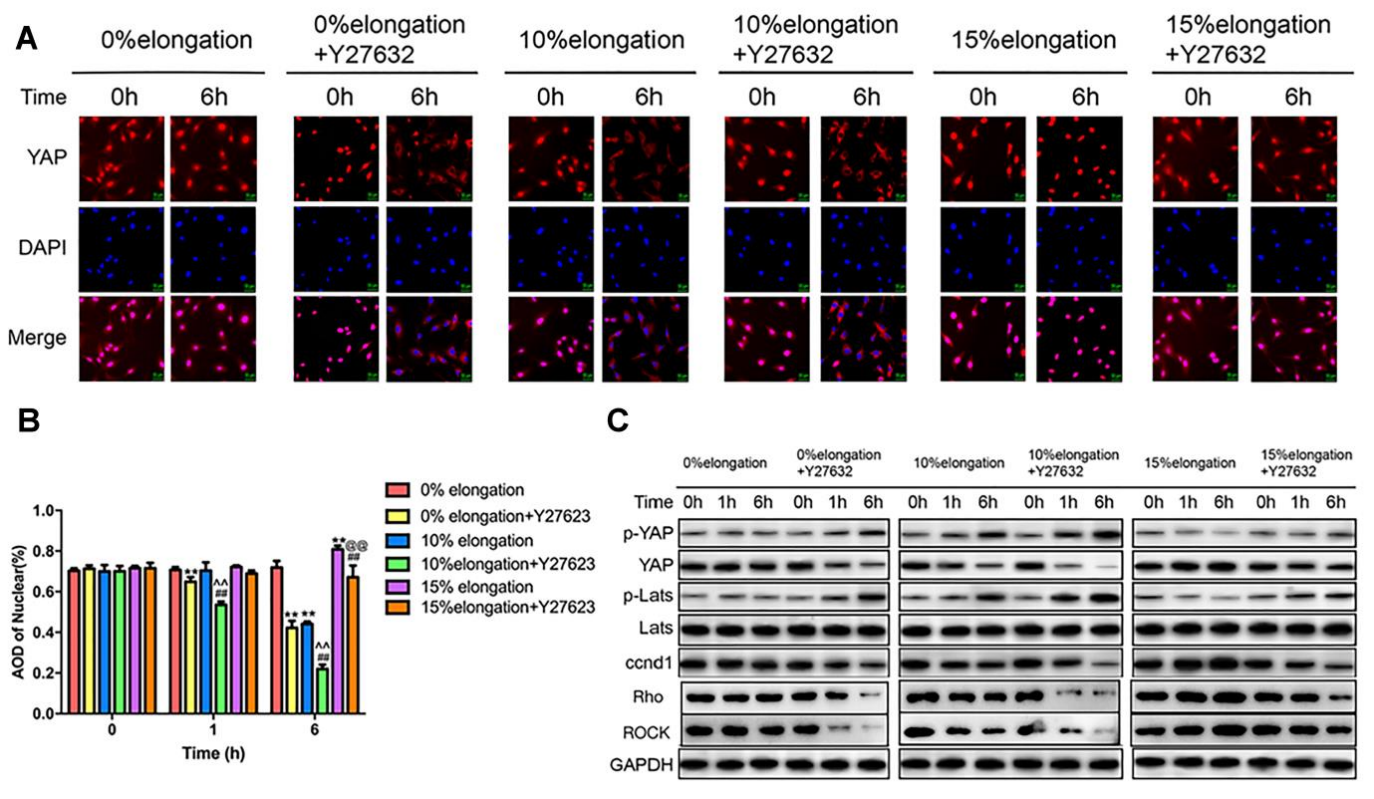
**Inhibition of the Rho-ROCK pathway affects the Hippo pathway under different intensities of stretch.** (**A**) The YAP localization of VSMCs was detected by immunocytochemistry. (**B**) Quantification of nuclear YAP amount in VSMCs. (**C**) The levels of phosphorylated YAP, phosphorylated Lats and ccnd1 in VSMCs were detected by western blotting. Values are expressed as means±SD. ^**^P < 0.01, compared with 0% elongation+siRNA(-) group; ^##^*P* < 0.01, compared with 10% elongation+siRNA(-) group; ^^^^*P* < 0.01, compared with 20% elongation+siRNA(-) group; ^@@^*P* < 0.01, compared with 0% elongation+siRNA(+) group.

To better understand the role of the Rho-ROCK-Hippo pathway in the effects of mechanical stretch on YAP, we inhibited the Rho-ROCK pathway using Y27632. We performed immunocytochemistry staining to determine the intracellular localization of YAP. The results showed that more YAP stayed in the cytoplasm under 0% elongation following inhibition of the Rho-ROCK pathway (group 2) compared with 0% elongation (group 1) (Figure 4A, 4B). Y27632 treatment combined with 10% elongation (group 4) caused a greater amount of YAP to remain in the cytoplasm compared with 10% elongation in the absence of Y27632 (group 3) (Figure 4A, 4B). The nuclear proportion of YAP following 15% elongation with Y27623 treatment (group 6) was higher than that in groups 2–4 (Figure 4A, 4B). The nuclear proportion in group 6 was lower than that in groups 1 and 5 (Figure 4A, 4B). We then performed western blotting to assess the phosphorylation of YAP and Lats1, as well as the protein levels of ccnd1, Rho, and ROCK in the different groups. The proportion of phosphorylated YAP and phosphorylated Lats1 increased under each stretch intensity following inhibition of the Rho-ROCK pathway, while the levels of ccnd1, Rho, and ROCK decreased, suggesting that blockage of the Rho-ROCK pathway partially reduces the effect of different stretch intensities on YAP (Figure 4C).

## DISCUSSION

**Figure 5:**
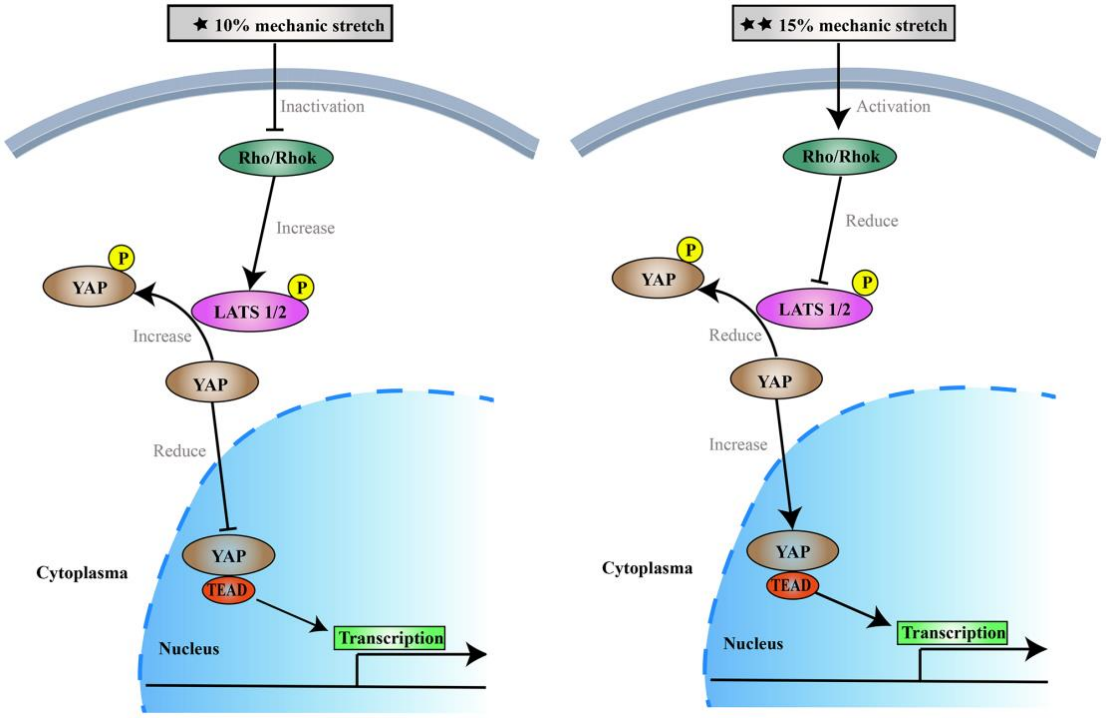
The effects of 10% and 15% elongation stretch on the proliferation and apoptosis of VSMCs.

One emerging concept is that changes in the VSMCs phenotype in response to mechanical forces are important in vascular diseases, such as hypertension, atherosclerosis, aortic aneurysms, and age-dependent arterial stiffening [21, 26, 27], in which the vascular wall is exposed to chronically elevated levels of cyclic stretch. In particular, the production of aortic aneurysms and aortic vascular VSMCs reduction are closely related. Different parts of the aorta are subject to different pressures, and the pathogenesis of aortic aneurysms may vary by location. Our study demonstrated that different intensities of stretch differently affect the proliferation and apoptosis of VSMCs. 10% and 20% of the stress-induced S phase cells reduce, may inhibit cell proliferation, 15% stress-stimulated S phase cells relative increase, may promote cell proliferation (Figure 5). However, we believe that VSMCs will not continue to grow, or may even break up, if more than 20% elongation force is applied. This also confirmed the effect of the difference of 10% physiological elongation and 15% stress elongation on the proliferation and apoptosis of VSMCs. Therefore, we believe that applying more tension to VSMCs is not necessary. In this study we sued 10% and 15% elongation forces, which we believe are more representative experimental conditions.

Studies have found that mechanical stimulation can inhibit the Hippo pathway, increase nuclear levels of YAP, promote cell proliferation, and inhibit apoptosis. [16, 28–31] YAP is the major downstream effector of the Hippo pathway which mediates major physiological functions. YAP phosphorylation results in cytoplasmic retention, which inhibits SMC proliferation and promotes apoptosis. [32, 33] To the best of our knowledge, however, this is the first study to show that different intensities of mechanical stretch can differentially influence the proliferation and apoptosis of VSMCs through the Hippo pathway. However, our study found that different intensities of mechanical stimulation had different effects on VSMCs’ proliferation and apoptosis, as well as the Hippo pathway. Stimulation with 10% elongation may activate the Hippo pathway, increasing phosphorylation of YAP and resulting in translocation of YAP into the cytoplasm, where it binds to the cytosolic protein 14-3-3 thereby promoting YAP degradation. These reports are consistent with our results. Our results revealed the intracellular localization of YAP under different stretch forces: 10% elongation for 6 h induced YAP cytoplasmic retention, while 15% elongation for 6 h promoted YAP entry into the nucleus. Mechanical stretching forces of 10% and 15% for 6 h upregulated and downregulated the phosphorylation of YAP, respectively. According to the former studies [34, 35], YAP in the nucleus, as well as the binding of YAP to the transcription factor TEAD, were decreased, leading to decreased expression of the target gene ccnd1 and inhibition of cell proliferation. When the stretching force was increased to 15%, the Hippo pathway was inhibited, YAP phosphorylation was reduced, more YAP remained in the nucleus, and the transcription factor TEAD induced expression of its downstream target gene ccnd1 to promote proliferation [36–38]. Thus, 10% and 15% stimulation can affect the proliferation of arterial SMCs through the Hippo pathway. The results demonstrate that 10% mechanical stimulation promotes cell proliferation and inhibits apoptosis, while 15% mechanical stimulation had the opposite effect. This trend is consistent with previous results on the Hippo pathway [36–38]. Previous studies have shown that YAP/TAZ expression in human trabecular meshwork cells also varies by substrate stiffness [39, 40]. The results of our study are consistent with previous studies investigating the effects of extracellular matrix on cells [41, 42]. After knocking down the expression of YAP, VSMCs showed the same trends in cell proliferation, apoptosis, and the cell cycle following stimulation with different intensities of stretch compared with the controls. These results indicated that different intensities of stretch stimulation could still regulate VSMCs proliferation and apoptosis following inhibition or activation of the Hippo pathway.

In addition, we tested the activity of the Rho-ROCK pathway and found that 10% elongation stimulated the expression of related proteins to decrease, while 15% elongation stimulated the expression of related proteins to increase. After blocking the Rho-ROCK pathway, the effects of different intensities of stretch on YAP were weakened and phosphorylated YAP was decreased in each group. Overall, inhibition of the Rho-ROCK pathway combined with 10% mechanical stress led to activation of the Hippo pathway, which in turn reduced nuclear localization of YAP. YAP failed to bind to TEAD; this led to a decrease in ccnd1 expression, inhibition of VSMCs proliferation, and promotion of apoptosis. When the mechanical stress was increased to 15%, we observed the opposite effects.

Through our experiments, we found that the mechanism underlying the effects of mechanical stress on VSMCs is as follows: after being stimulated by physiological 10% elongation stretch, VSMCs are inhibited in various ways. Inhibition of Rho-ROCK leads to a decrease in LATS1/2 levels, which in turn leads to an increase in the phosphorylation of YAP. Phosphorylated YAP cannot enter the nucleus nor bind to TEAD. On the other hand, our ongoing experiment shows that 10% elongation stretch may downregulates the expression of miR130a, which increases the amount of VGLL4. In the nucleus, VGLL4 may competes with YAP for the opportunity to bind TEAD. As a result of these two mechanisms, 10% elongation reduced ccnd1, resulting in decreased VSMCs proliferation and increased apoptosis, while a stimulus that is lower or higher than physiological elongation resulted in opposing effects, eventually promoting the proliferation of VSMCs and reducing apoptosis.

On conclusion, our study demonstrates the possibility of preventing or treating diseases such as aortic aneurysms by controlling blood pressure. The Rho-ROCK pathway can be activated by mildly adjusting the blood pressure. Activation of the Rho-ROCK pathway, in turn, inhibits the Hippo pathway and ultimately promotes VSMCs proliferation and inhibits apoptosis. Our findings may provide insight into the pathogenesis prognosis of these diseases, as well as into therapeutic interventions.
